# Hemodynamic changes and perinatal outcome associated with umbilical artery thrombosis: a retrospective study

**DOI:** 10.1186/s13023-024-03107-y

**Published:** 2024-03-05

**Authors:** Peng Tu, Xiaohang Zhang, Chunyan Zhong, Qian Ran, Suzhen Ran

**Affiliations:** 1 Department of Ultrasound, Women and Children’s Hospital of Chongqing Medical University, 401147 Chongqing, China; 2 Department of Ultrasound, Chongqing Health Center for Women and Children, 401147 Chongqing, China

**Keywords:** Umbilical artery thrombosis, Hemodynamic changes, Perinatal outcomes

## Abstract

**Objective:**

Poor fetal and perinatal outcomes in fetuses associated with umbilical artery thrombosis (UAT), such as severe intrauterine growth restriction (IUGR) and intrauterine asphyxia have been reported by some case series. Its hemodynamic impact remains unclear. The aim of this study was to evaluate the hemodynamic changes and perinatal outcome in UAT fetuses with a relatively large sample.

**Methods:**

We included singleton fetuses diagnosed with UAT and with at least one available Doppler evaluation before the end of pregnancy in our center from 2016 to 2023. Fetuses with structural abnormalities and with no follow-up results were excluded. Doppler waveforms from the Umbilical artery (UA), middle cerebral artery (MCA), ductus venosus (DV) and uterine artery (UtA) were routinely evaluated according to ISUOG Practice Guidelines from diagnosis. The same sample of GA-matched normal fetuses with Doppler measurements during the same period were randomly selected as control group.

**Results:**

Eighty-nine singleton fetuses with UAT with at least one Doppler evaluation before the end of pregnancy were identified, 13 fetuses with no follow-up results were excluded. After comprehensive prenatal counseling, 14 cases received urgent cesarean section, the remaining 55 cases received expectant management, the median day between GA at diagnosis and end of pregnancy was 13 (5–53) days (range, 2-159). 7 (7/76, 9.2%) cases occurred stillbirth, and the incidence of IUGR and Neonatal Intensive Care Unit (NICU) admission were 18.4% (14/76) and 13.2% (10/76) respectively. 49 fetuses (49/76, 64.5%) combined with Doppler abnormalities. UA abnormalities (35/76, 46.1%) and MCA abnormalities (34/76, 44.7%) were the most changes at presentation. Compared to control group, UA-EDV was significantly increased in UAT fetuses [21.84 (15.59–26.64) vs. 16.40 (12.43–20.70) cm/s, *p* < 0.001], UA-PI and UA-RI significantly decreased [0.68 (0.57–0.84) vs. 0.92 (0.79–1.11), *p*<0.001; 0.51 (0.44–0.59) vs. 0.62 (0.55–0.68), *p* < 0.001, respectively]. Both the MCA-PSV and MCA-EDV were significantly higher in UAT fetuses [54.60 (48.00-61.34) vs. 44.47 (29.66–57.60) cm/s, *p* < 0.001; 11.19 (7.84–17.60) vs. 8.22 (5.21-12.00) cm/s, *p* < 0.001, respectively], this led to a lower MCA-PI and MCA-RI. Meanwhile, DV-PIV was significantly higher in UAT fetuses [0.6 (0.47–0.87) vs. 0.45 (0.37–0.55), *p <* 0.001], CPR and UtA-PI were no significant difference between these two groups. Multivariate logistic regression analysis showed that DV-PIV was an independent risk factor for adverse pregnancy outcomes (OR 161.922, *p*<0.001), the area under the ROC curve (AUC) was 0.792 (95% CI 0.668–0.917; *p* < 0.001).

**Conclusion:**

Our data showed serious adverse pregnancy consequences are combined with UAT fetuses. Hemodynamic changes in UAT fetuses showed the remaining artery for compensation and brain perfusion derangement. With a comprehensive and standardized Doppler evaluation, progression of fetal deterioration may be detailed presented.

**Supplementary Information:**

The online version contains supplementary material available at 10.1186/s13023-024-03107-y.

## Introduction

Umbilical artery thrombosis (UAT) is rare and associated with serious complications, such as intrauterine growth restriction (IUGR), unpredictable intrauterine distress, or even stillbirth [1, 2]. Case reports with UAT mainly focused on etiology analysis, clinical interventional procedures and prediction model [3–5]. Due to fetal occlusion or unclear previous pregnancy history, UAT is easy to miss diagnosis. As a consequence, our understanding of adaptive processes in hemodynamic and the ability to predict adverse pregnancy outcomes remain weak.

Due to acute hemodynamic derangement, UAT is associated with significant mortality [6]. In order to avoid adverse outcomes, inevitable cesarean section and iatrogenic premature birth may occurred after the diagnosis [7]. Detecting the hemodynamic changes of UAT during pregnancy remains a challenge, and how to prolong pregnancy and monitor intrauterine growth is an urgent problem to be solved.

The “utero-placental circulation” (uterine artery), “placenta-fetal circulation” (umbilical artery), and “fetal circulation” (middle cerebral artery, ductus venosus) are three important pathways in the fetal period [8]. The usefulness of umbilical artery (UA) and middle cerebral artery (MCA) in the assessment of umbilical cord and brain circulation has been established in extensive researches. The results showed that the redistribution of blood greatly influence the development of fetal nervous system in perinatal period [9, 10].

The focus of this study is to present the hemodynamic changes in UAT fetuses with a relatively large sample. We also described the differences of hemodynamic data between fetuses with UAT and gestational-age (GA) matched controls, with an emphasis on quantitative indices of blood flow redistribution before the end of pregnancy. Such findings may not only present intuitive hemodynamic data for obstetricians and sonographers in UAT fetuses, but also provide reliable basis for clinical intervention and parental counseling.

## Methods

### Study participants

This was a retrospective study including singleton fetuses diagnosed with UAT from January, 2016 to June, 2023 at Women and Children’s Hospital of Chongqing Medical University. Our hospital is an academic tertiary referral care center, accepting the high-risk pregnant women in Southwest China. A total of 140,771 fetuses referred to our center underwent a routine second-trimester ultrasound examination.

We systematically analyzed the cases retrieved as " umbilical artery thrombosis " in the medical information system. Diagnostic clue for UAT: (1) according to the Guildlines [11], color flow mapping should be used to identify the image of the bladder with the two umbilical arteries in the first or earlier second trimester; (2) color Doppler flow present an absent paravesical during the next second-trimester ultrasound examination, and two-dimensional ultrasound show one umbilical artery and one vein in the cross section of umbilical cord with hyperechoic thromboembolism in part of the UA.

Further inclusion criteria for UAT: (1) Singleton pregnancy; (2) after the identification of UAT, at least one fetal biometry and Doppler measurements available for review; (3) absence of structural and chromosomal abnormalities in perinatal period; (4) complete perinatal and neonatal follow-up materials. Exclusion criteria: (1) Multiple pregnancy; (2) UAT fetuses with incomplete clinical data; (3) delivery at other medical centers.

The same sample of GA-matched normal fetuses with Doppler measurements during the same period were randomly selected as control group. Inclusion criteria for control group: (1) None of the control fetuses had chromosomal or structural abnormalities; (2) pregnant women with no serious complications. The reason for the Doppler examination was the pregnant woman’s suspicion of reduced fetal movement.

Pregnancies were dated according to the last menstrual period (LMP) and confirmed by crown-rump length (CRL) measurements at 11-14week nuchal translucency scan. The study was approved by the Ethics Committee of Women and Children’s Hospital of Chongqing Medical University and all pregnant women gave written consents.

### Ultrasound and doppler examinations

After the diagnosis of UAT, Doppler waveforms from the UA, MCA, ductus venosus (DV) and uterine artery (UtA) of UAT and control fetuses were routinely evaluated. Ultrasound and Doppler measurements were performed using the Voluson E8/E10 system (GE Healthcare Ultrasound, Milwaukee, WI, USA) or Philips iU22 (Phillips Medical systems, Bothell, WA) with probe frequency of 2-8 MHz. All two-dimensional and Doppler examination were performed in line with the ISUOG Practice Guidelines [12] and measured by two experienced physicians (X.H.Z and S.Z.R). The standardized procedure of Doppler measurement of fetuses has been reported [12]: (1) Doppler recordings were obtained in the absence of fetal movements and during temporary maternal breath-holding; (2) the insonation was aligned with the direction of blood flow, which was closely to 0°; (3) a low vessel wall filter was used to record low diastolic flow.

Peak systolic velocity (PSV), end-diastolic velocity (EDV) of UA, MCA and UtA were estimated by the system software, the commonly used Doppler indices, i.e. pulsatility (PI), resistance (RI) indices in UA and MCA were calculated. Pulsatility index (PI) was calculated as peak systolic velocity minus diastolic velocity divided by the mean velocity, resistance index (RI) was calculated as peak systolic velocity minus diastolic velocity divided by peak systolic velocity.

The cerebroplacental Doppler ratio (CPR) was calculated as the PI of MCA divided by the PI of UA. Pulsatility index for veins (PIV) was used for analysis of the DV, which was calculated as PIV=(Vs − Va)/TAMX, where Vs is the peak forward velocity during ventricular systole and Va is the lowest forward velocity or peak reversed velocity during atrial contraction (the ‘a-wave’), and TAMX is the maximum velocity.

To investigate the specific distribution of hemodynamic in UAT fetuses, all parameters were compared with reference indexes for gestation (<5th centile or>95th centile for gestational age were defined as abnormalities), and the distribution of blood flow abnormalities were categorized. IUGR was defined as guidelines by an estimated fetal weight below the 10th centile for GA, with or without Doppler abnormalities (one or more of the following: UA PI > 95th centile, UtA PI > 95th percentile, CPR < 5th centile) [13].

### Management

In order to avoid inevitable cesarean section and iatrogenic premature birth, the management were mainly based on fetal hemodynamic, electronic heart rate monitoring and GA. After comprehensive prenatal counseling, pregnant woman received urgent cesarean section or expectant management. In expectant management group, pregnant women were strictly required to have electronic heart monitoring every 2 days, ultrasound hemodynamic evaluation once a week, and closely monitored fetal movement. Low molecular weight heparin (LMWHs) and oxygen inhalation were used as appropriate, and dexamethasone was given to accelerate fetal lung maturation.

### Pathological examination and follow-up

Gross examination of the umbilical cord (UC), including the length and spiral number, were recorded. The UC abnormalities were excessively long cord (100 cm or beyond), excessively short cord (30 cm or less), and torsion of the cord (11 circles or more) [14]. The diagnosis of UAT was confirmed by microscopic.

Clinical characteristics were obtained through the medical record systems, the following items were analyzed: (1) maternal age, body mass index (BMI), maternal complications (including gestational diabetes mellitus (GDH), gestational hypertension (GH), chronic thyroid disease and coagulation function abnormalities; (2) data of fetuses and neonates, including UC abnormalities, prenatal heart monitoring, delivery mode and adverse perinatal outcomes.

### Statistical analysis

With regard to statistical analysis, variables were tested for Shapiro-wilk normal distribution, and described using mean and standard deviation (SD) or median and range, as appropriate. Student’s *t*-test, Mann-Whitney *U* test and *Chi*-square test were used to compare continuous variables between UAT fetuses and controls. Univariate and multivariable logistic regression models were performed to explore relevant risk factors. Receiver operating characteristic (ROC) curves were constructed to evaluate the diagnostic value of the parameters. A *P*-value < 0.05 was considered statistically significant. Data were analyzed by the software SPSS statistical for Windows version 22.0.

## Results

### Clinical characteristics

Eighty-nine singleton fetuses with UAT were identified, which represent a prevalence of 0.06% (89/140,771) in our center. Thirteen fetuses with no follow-up results were excluded, yielding 76 singleton fetuses were eligible for the analysis in the study. Differences in clinical characteristics and Doppler parameters in the UAT fetuses and control groups were presented in Table 1. The mean maternal age was 29.80 ± 4.23 years and median GA at diagnosis was 30^6/7^ (25–35^2/7^) weeks (range,15^6/7^-38^4/7^). For control group, the mean maternal age was 30.00 ± 3.83 years and median GA at diagnosis was 33 (24–36^5/7^) (range, 21^6/7^-40^1/7^) weeks.

**Table 1:** Differences in clinical characteristics and doppler parameters in the umbilical artery thrombosis (UAT) fetuses and control groups

Variable	UAT (*n* = 76)	Control (*n* = 76)	P-value
Clinical characteristics			
Maternal age (years)	29.80 ± 4.23	30.00 ± 3.83	0.764
BMI (kg/m^2^)	21.16 (19.58–3.04)	21.49 (19.23–23.33)	0.930
GA at diagnosis (weeks)	30.6 (25.0-35.2)	31.2 (24.0-36.5)	0.191
GA at delivery (weeks)	36.1 (32.2–38.1)	38.7(38.3–39.3)	<0.001
GDM	17/76 (22.4)	0 (0.0)	<0.001
GH	11/76 (14.5)	2/76 (2.6)	0.009
Chronic thyroid disease	5/76 (6.6)	0 (0.0)	0.023
Coagulation function abnormalities	3/76 (3.9)	0 (0.0)	0.081
Abnormal amniotic fluid	12/76 (15.8)	0 (0.0)	<0.001
Birth weight (g)	2430 (1625–2975)	3163.13(2892–3345)	<0.001
Apgar 1-min	9.1 (10.0, 10.0)	10 (10, 10)	<0.001
Apgar 5-min	9.2 (10.0, 10.0)	10 (10, 10)	0.004
UC abnormalities	28/76 (36.8)	3/76 (3.9)	<0.001
Cesarean section	59/76 (77.6)	33/76 (43.4)	<0.001
Doppler parameters			
UA- PSV (cm/s)	43.40 (34.90-53.18)	43.05 (35.34–49.82)	0.704
UA- EDV (cm/s)	21.84 (15.59–26.64)	16.40 (12.43–20.70)	<0.001
UA-PI	0.68 (0.57–0.84)	0.92 (0.79–1.11)	<0.001
UA-RI	0.51 (0.44–0.59)	0.62 (0.55–0.68)	<0.001
MCA- PSV (cm/s)	54.60 (48.00-61.34)	44.47 (29.66–57.60)	<0.001
MCA- EDV (cm/s)	11.19 (7.84–17.60)	8.22 (5.21-12.00)	<0.001
MCA-PI	1.29 (0.98–1.47)	1.71 (1.56–1.89)	<0.001
MCA-RI	0.79 (0.66–0.85)	0.81 (0.78–0.84)	0.047
CPR	1.84 (1.45–2.36)	1.84 (1.60–2.20)	0.704
DV-PIV	0.6 (0.47–0.87)	0.45 (0.37–0.55)	<0.001
UtA-PI	0.63 (0.52–0.72)	0.66 (0.55–0.76)	0.090

Data are given as mean ± SD, n (%) or median (interquartile range). *P*-values calculated using *t*-test or *χ2* test, as appropriate. BMI, body-mass index; GA: gestational age; GDM, gestational diabetes mellitus; GH, gestational hypertension; Umbilical cord (UC) abnormalities: excessive long cord, short cord, true knots and torsion of the cord; UA, umbilical artery; PSV, peak systolic velocity; EDV, end-diastolic velocity; PI, pulsatility indices; RI, resistance indices; MCA, middle cerebral artery; CPR, cerebroplacental doppler ratio; DV, ductus venosus; PIV, pulsatility index for veins; UtA, uterine artery

As shown, the incidence of GDM, GH and chronic thyroid disease in the UAT group were significantly higher than those in the control group. However, no differences were reported for maternal age, BMI, GA at diagnosis and the incidence of coagulation function abnormalities. (Table 1).

After comprehensive prenatal counseling, 14 cases received urgent cesarean section, the mean GA at diagnosis was 34^3/7^ ± 3^6/7^ weeks. Stillbirth occurred in 7 cases, the mean GA at diagnosis was 24^5/7^ ± 1^6/7^ weeks. The remaining 55 cases received expectant management, the median GA at diagnosis was 31 (25–35^2/7^) weeks (range, 15^6/7^-38^4/7^), and the median day between GA at diagnosis and end of pregnancy was 13 (5–53) days (range, 2-159).

### Doppler findings

**Fig. 1:**
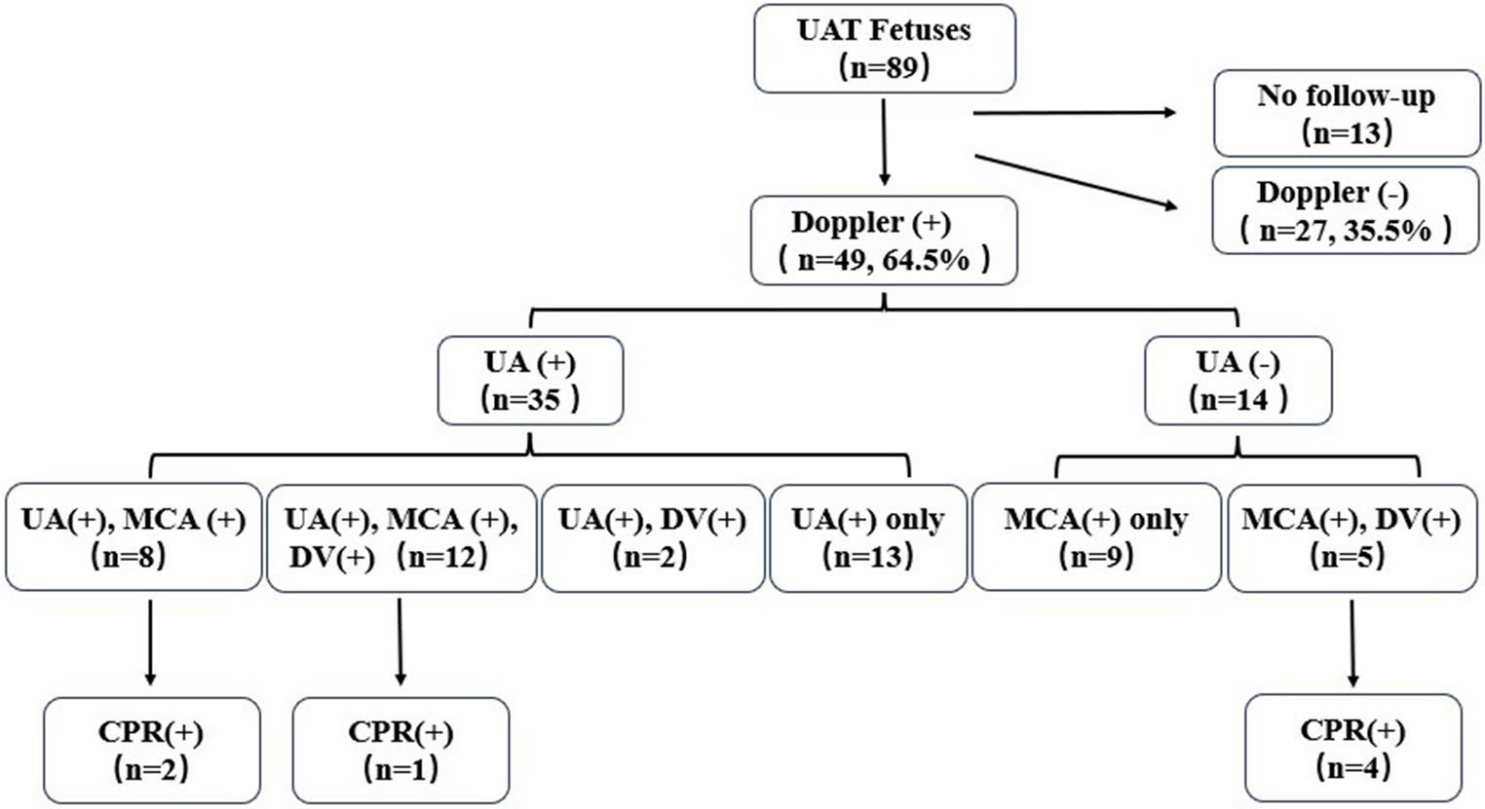
The specific distribution of hemodynamics in UAT fetuses

**Fig. 2:**
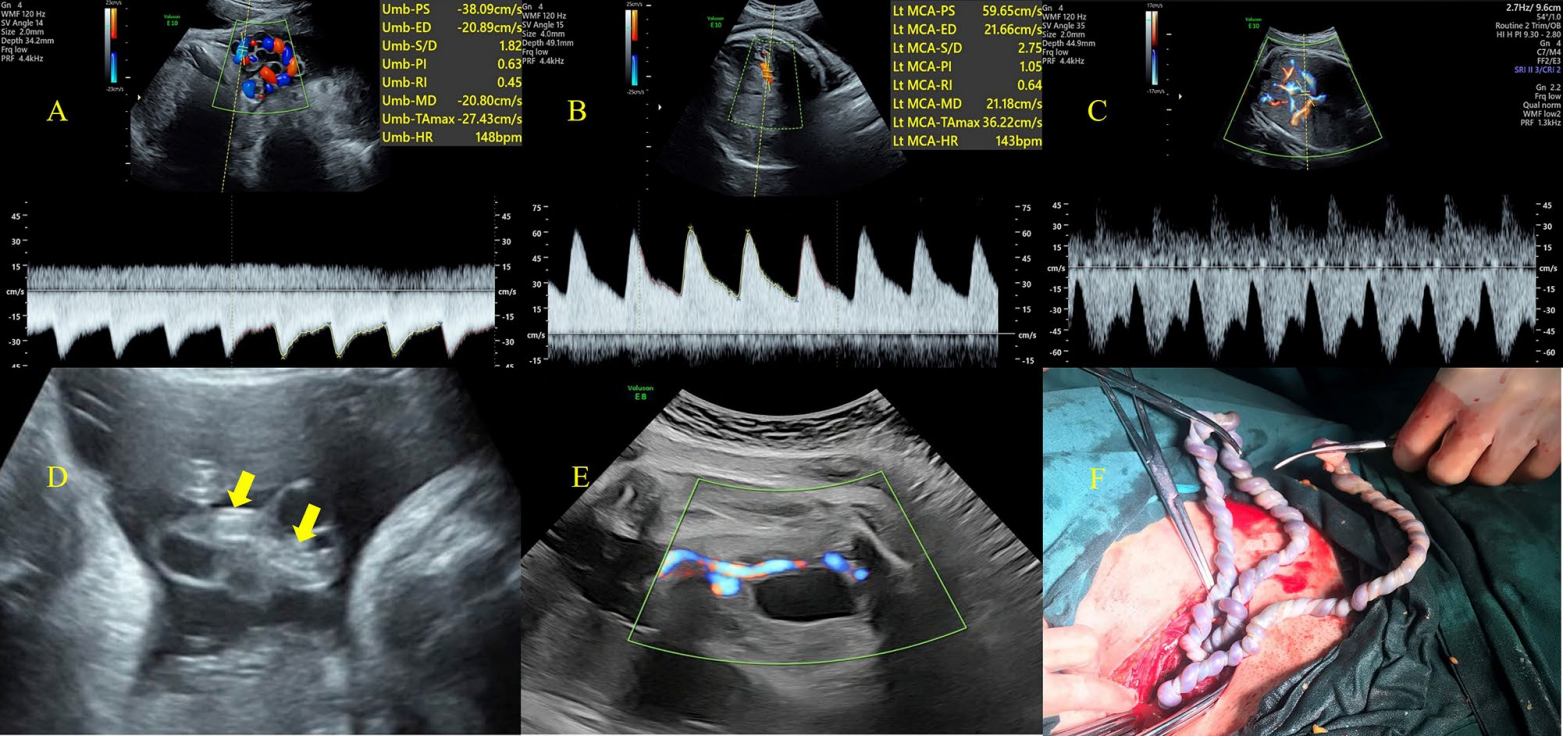
Hemodynamics arrangement of the UAT fetuses (GA at diagnosis: 36^1/7^ weeks). **A** Waveforms from UA, the value of S/D and PI were both abnormal (<5th centile for gestational age). **B** Waveforms from MCA, the value of PI was abnormal (<5th centile for gestational age). **C** Ductus venosus Doppler recording showed the ‘a-wave’, which was close to the zero line. **D**, Hyperechoic thromboembolism (yellow arrow) was observed in a part of the UA through two-dimensional ultrasound. **E** Colour Doppler flow showed only one umbilical artery in the section of bladder. **F** Excessive torsion of umbilical cord presented by gross observation

Figures 1 and 2 showed the specific distribution of hemodynamics in UAT fetuses, 49 fetuses (49/76, 64.5%) combined with Doppler abnormalities. The most changes at presentation were UA abnormalities (35/76, 46.1%) and MCA abnormalities (34/76, 44.7%). Seven fetuses (7/76, 9.2%) were combined with CPR abnormalities and 19 fetuses (19/76, 25%) presented with right heart dysfunction (DV-PIV>95th centile).

In order to explore the quantitative indicators which can reflect the hemodynamic changes of UAT, we compared associated parameters (Table 1). In UA hemodynamic changes, UA-PSV was no difference between UAT fetuses and control group; UA-EDV was significantly increased in UAT fetuses [21.84 (15.59–26.64) vs. 16.40 (12.43–20.70) cm/s, *p <* 0.001], UA-PI and UA-RI significantly decreased as compared to controls [0.68 (0.57–0.84) vs. 0.92 (0.79–1.11), *p <* 0.001; 0.51 (0.44–0.59) vs. 0.62 (0.55–0.68), *p <* 0.001, respectively].

In terms of fetal cerebral blood flow redistribution, MCA blood flow changed significantly, as depicted in Table 1. Both the MCA-PSV and MCA-EDV were significantly higher in UAT fetuses [54.60 (48.00-61.34) vs. 44.47 (29.66–57.60) cm/s, *p <* 0.001; 11.19 (7.84–17.60) vs. 8.22 (5.21-12.00) cm/s, *p <* 0.001, respectively]. This led to a lower MCA-PI and MCA-RI in UAT fetuses. Compared with the control group, DV-PIV was significantly higher in UAT fetuses [0.6 (0.47–0.87) vs. 0.45 (0.37–0.55), *p <* 0.001]. However, CPR and UtA-PI were no significant difference between these two groups.

### Pregnancy outcomes and follow-up

Fetal heart tracing was performed on 65 pregnant women in UAT group, the minority of pregnant women (10/65, 15.4%) had non contractile stimulation test (NST) abnormalities. During the management, LMWHs was used in the treatment of 7 pregnant women, the indications were 1 case of thrombophilia, 1 case of non-obstetric antiphospholipid syndrome (NC-OAPS), 2 cases of FGR, 1 case of FGR with NC-OAPS, 1 case of FGR with thrombophilia, and 1 cases of empirical use of LMWH to improve placental circulation.

Intrauterine death occurred in 7 (7/76, 9.2%) cases, and the incidence of IUGR and Neonatal Intensive Care Unit (NICU) admission were 18.4% (14/76) and 13.2% (10/76) respectively. The median GA at delivery and birth weight were more decreased in UAT fetuses. Meanwhile, the rate of cesarean section in UAT fetuses (59/76, 77.6%) was significantly higher than those in the control group. Apgar 1-min and Apgar 5-min were significant difference between these two groups (Table 1). The placenta and umbilical belt were routinely examined, 28 fetuses (28/76, 36.8%) with UC overtorsion. Thirty-one patients were selected for placental pathology examination, UAT was histologically confirmed.

**Fig. 3:**
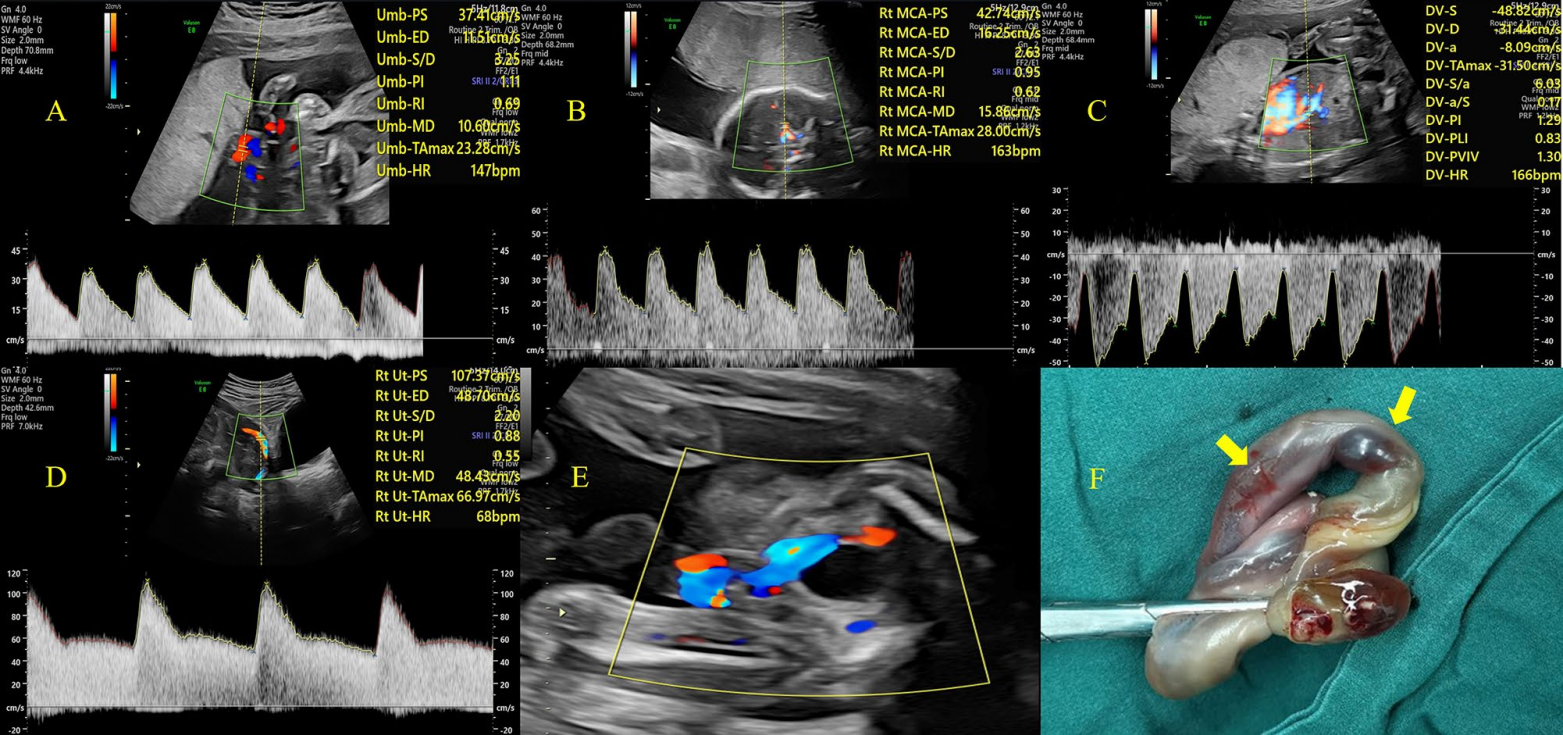
Hemodynamics arrangement of the UAT fetuses with stillbirth (Case 2 in Table 3). **A** Waveforms from UA, Doppler parameters were both within normal range. **B** Waveforms from MCA, the value of PI was abnormal (<5th centile for gestational age). **C** Ductus venosus Doppler recording showed the ‘a-wave’, the value of PIV was abnormal (> 95th centile for gestational age). **D** Waveforms from UtA, Doppler parameters were both within normal range. **E** Colour Doppler flow showed only one umbilical artery in the section of bladder. **F** Section through the umbilical cord: thromboembolism in one UA, dark brown appearance (yellow arrow)

Table 2 summarized the Doppler characteristics of the seven cases with stillbirth. The mean GA at end of pregnancy was 25^2/7^ ± 2^1/7^ weeks (range, 23^4/7−^28^1/7^), and the mean time between GA at diagnosis and end of pregnancy was 4^3/7^ ± 2^4/7^ days (range, 1–8). In the Doppler findings (Fig. 3), two fetuses (2/7, 28.6%) with UA abnormalities: UA-PI < 5th centile and UA-PI < 5th centile; seven fetuses (7/7, 100.0%) with MCA-PI < 5th centile. Six fetuses (6/7, 85.7%) with CPR < 5th centile; seven fetuses (7/7, 100.0%) with right atrial dysfunction: DV-PIV>95th centile.

**Table 2:** Doppler parameters of umbilical artery thrombosis (UAT) with stillbirth

Case	GA at diagnosis of UAT (weeks)	GA at end of pregnancy (weeks)	Time between GA at diagnosis and termination of pregnancy (days)	UA-PI	UA-RI	MCA-PI	CPR	DV-PIV	UtA-PI
1	24^4/7^	25^3/7^	5	1.25	0.77	0.85^a^	0.68^a^	1.10^c^	0.74
2	23^2/7^	24	4	1.11	0.69	0.95 ^a^	0.91 ^a^	1.29 ^c^	0.88
3	26^5/7^	28^1/7^	8	0.73 ^a^	0.54 ^a^	1.38 ^a^	1.89 ^b^	1.14 ^c^	0.56
4	22^5/7^	23^4/7^	5	0.78 ^a^	0.56 ^a^	0.89 ^a^	1.14 ^a^	1.15 ^c^	0.45
5	27	27^2/7^	2	0.83^b^	0.59 ^b^	0.69 ^a^	0.84 ^a^	0.89 ^c^	0.58
6	24^1/7^	24^2/7^	1	0.93 ^b^	0.63 ^b^	1.10 ^a^	1.18 ^a^	1.10 ^c^	0.47
7	24	24^2/7^	2	1.01 ^b^	0.67 ^b^	0.81 ^a^	0.80 ^a^	1.16 ^c^	0.75

UA, umbilical artery; PI, pulsatility indices; RI, resistance indices; MCA, middle cerebral artery; CPR, cerebroplacental doppler ratio; DV, ductus venosus; PIV, pulsatility index for veins; UtA, uterine artery

a,<5th centile; b, 5th -50th centile; c, >95th centile

**Table 3:** Doppler parameters of umbilical artery thrombosis (UAT) with intrauterine growth restriction (IUGR)

Case	GA at diagnosis of UAT (weeks)	GA at end of pregnancy (weeks)	Time between GA at diagnosis and termination of pregnancy (days)	Present time of IUGR	Pregnancy Outcomes	UA-PI	UA-RI	MCA-PI	CPR	DV-PIV	UtA-PI
1	28^2/7^	30^5/7^	17	meanwhile	(-)	0.90	0.62	0.91^a^	1.01	0.44	0.60
2	28^5/7^	38^5/7^	70	meanwhile	(-)	0.60	0.46	1.24	2.05	0.62	0.81
3	33	34^1/7^	8	meanwhile	(-)	0.99	0.66	1.53	1.55	0.52	0.52
4	35^2/7^	36	5	meanwhile	(-)	0.63 ^a^	0.48 ^a^	1.29 ^a^	2.04	1.03 ^b^	0.62
5	32	34	14	meanwhile	(-)	0.88	0.61	1.59	1.82	0.52	0.66
6	32	32^2/7^	2	meanwhile	(-)	0.46 ^a^	0.38 ^a^	1.06 ^a^	2.73	0.7	0.42
7	34^1/7^	35	6	meanwhile	(-)	0.60 ^a^	0.46 ^a^	1.46	2.42	0.38	0.49
8	29^3/7^	32	18	meanwhile	(-)	0.76	0.55	1.17 ^a^	1.54	0.74	0.63
9	24^4/7^	25^3/7^	6	meanwhile	stillbirth	1.25	0.77	0.85 ^a^	0.68 ^a^	1.1 ^b^	0.74
10	23^2/7^	24	5	meanwhile	stillbirth	1.06	0.69	0.96 ^a^	0.91 ^a^	1.29 ^b^	0.55
11	22^5/7^	23^4/7^	6	meanwhile	stillbirth	0.78 ^a^	0.56 ^a^	0.89 ^a^	1.14	1.15 ^b^	0.45
12	24	24^2/7^	2	meanwhile	stillbirth	1.01	0.67	0.81 ^a^	0.8 ^a^	1.16 ^b^	0.73
13	31	39	56	45 days after the diagnosis of UAT	(-)	0.53 ^a^	0.42 ^a^	1.39	2.64	0.74	0.65
14	27^5/7^	35^5/7^	56	30 days before the diagnosis of UAT	(-)	0.55 ^a^	0.43 ^a^	1.32 ^a^	2.38	0.68	0.54

UA, umbilical artery; PI, pulsatility indices; RI, resistance indices; MCA, middle cerebral artery; CPR, cerebroplacental doppler ratio; DV, ductus venosus; PIV, pulsatility index for veins;

UtA, uterine artery. ^a^,<5th centile; ^b^,>95th centile. (-): No adverse perinatal outcomes occurred

The Doppler characteristics of UAT with IUGR presented in Table 3 separately. A total of 14 UAT fetuses were associated with IUGR, 12 of which were detected at the same stage with diagnosis, one was 45 days after diagnosis of UAT and one was 30 days before diagnosis of UAT. The mean GA at diagnosis was 29 ± 4^2/7^ weeks, and the mean GA at end of pregnancy was 32 ± 5^4/7^ weeks.

### Prediction of the risk factors of adverse pregnancy outcomes

We defined adverse pregnancy outcomes as intrauterine death, IUGR and NICU admission. The univariate logistic regression analysis showed that MCA-PI, CPR and DV-PIV were risk factors for adverse pregnancy outcomes (*p =* 0.007, 0.023,<0.001). The multivariate logistic regression analysis showed that DV-PIV was an independent risk factor for adverse pregnancy outcomes (OR 161.922, *p*<0.001) (Table 4).

**Table 4:** Prediction of the risk factors of adverse pregnancy outcomes

	Univariate		Multivariate	
	OR (95% CI)	p	OR (95% CI)	p
Maternal age	1.028 (0.919–1.149)	0.630		
GA at diagnosis	0.979 (0.903–1.062)	0.609		
UC abnormalities	1.580 (0.612–4.084)	0.345		
Abnormal amniotic fluid	0.520 (0.128–2.108)	0.360		
UA-PI	2.842 (0.376–21.515)	0.312		
MCA-PI	0.087 (0.015–0.508)	0.007	1.512 (0.086–26.506)	0.777
CPR	0.392 (0.175–0.879)	0.023	0.394 (0.106–1.469)	0.165
DV-PIV	176.759 (16.244-1923.438)	<0.001	161.922 (12.166-2155.123)	<0.001
UtA-PI	0.725 (0.024–21.978)	0.854		

GA, gestational age; UC, Umbilical cord; UA, umbilical artery; PI, pulsatility indices; MCA, middle cerebral artery; CPR, cerebroplacental doppler ratio; DV, ductus venosus; PIV, pulsatility index for veins; UtA, uterine artery. Adverse pregnancy outcomes included stillbirth, intrauterine growth restriction (IUGR) and Neonatal Intensive Care Unit (NICU) admission

**Fig. 4:**
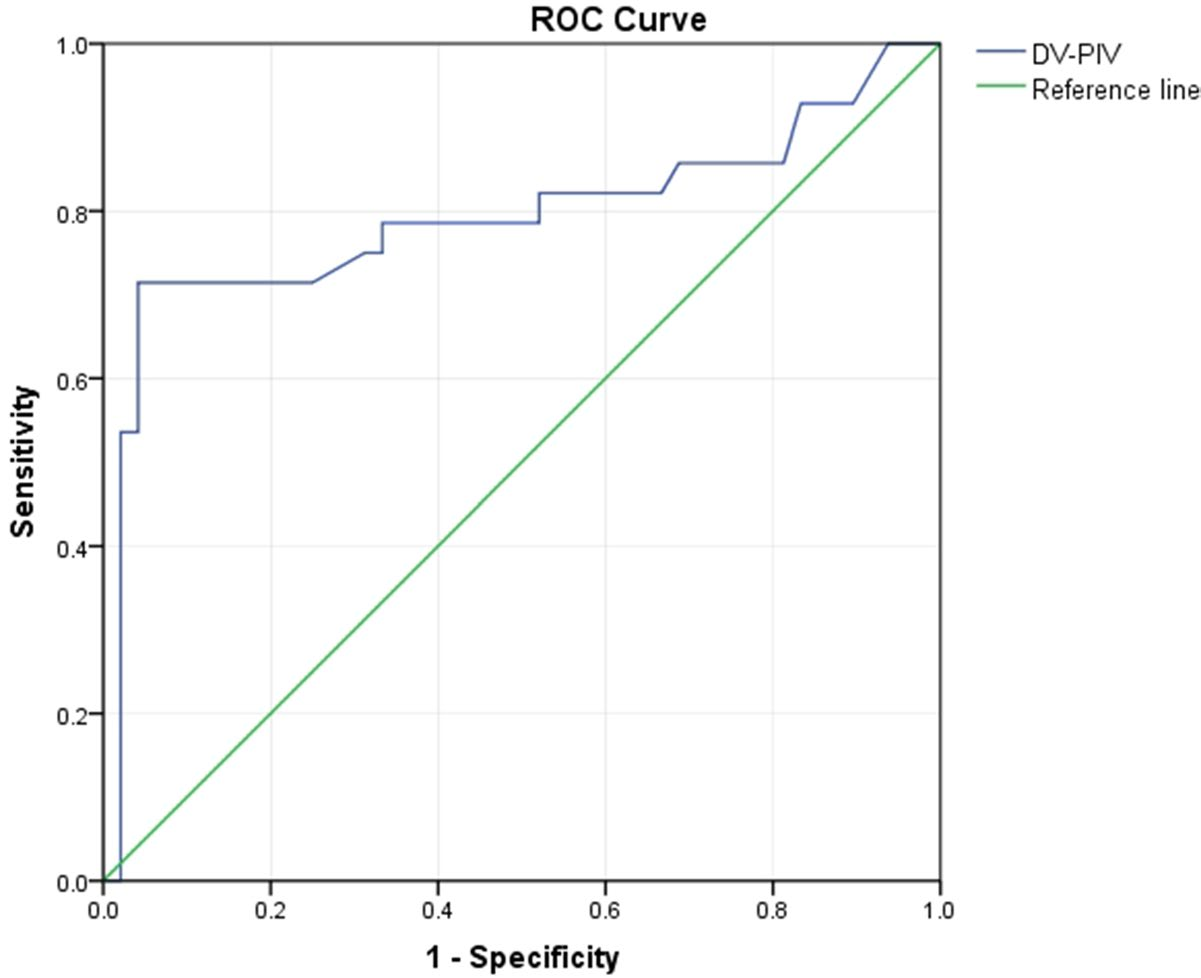
ROC Curve. Receiver operating characteristic (ROC) analysis was used to evaluate the prediction of the risk factors of adverse pregnancy outcomes. DV, ductus venosus; PIV, pulsatility index for veins

Meanwhile, we analyzed the prediction performance of DV-PIV by ROC curves based on the results of the regression analysis (Fig. 4). DV-PIV had moderate predictive accuracy for adverse pregnancy outcomes (AUC [95% CI] 0.792 [0.668–0.917]; *p* < 0.001). The result showed that UAT fetuses with a DV-PIV value more than 0.76 tended to have adverse pregnancy outcomes (Youden’s index was 0.673), with sensitivity of 71.4% and specificity of 95.8%.

## Discussion

Although individual case reports and clinical outcome analysis were available [15], the hemodynamic features of the fetuses with UAT have not been well delineated. The incidence of UAT is 0.025–0.045% [16], which is lower than the incidence rate in our unite. In case series by Wei et al. [7] the incidence of UAT was 0.8%, probably due to both our center were tertiary referral care center.

*The pathogenesis of UAT.* The pathogenic factors of UAT are still unclear, previous findings showed that slow blood flow in the umbilical cord, coursed by cord knotting or twisting, vascular endothelial inflammation and maternal coagulation abnormalities may be the main factors causing UAT [17]. Jiang et al. [18] demonstrated that the incidence of UC torsion is 19%. In our study, UC abnormalities were present in a large proportion (36.8%), which was measured after delivery.

In addition to the UC abnormalities, maternal complications such as GDH, GH, coagulation function abnormalities and other factors may also be the pathogenic factors for UAT. Wu et al. [5] presented that GDM was the most frequent concomitant disease (20%) associated with UAT. Our data of clinic characteristics showed that the percent of GDM and GH was significantly higher than the control group. The reason may be that hypertension and hyperglycemia induce the imbalance of endothelial vascular factor expression and endothelial injury, leading to coagulation dysfunction and thrombosis.

*Hemodynamic arrangement of UAT.* The “early warning signal” of the fetus can be observed by monitoring the changes of fetal hemodynamics through Doppler velocimetry, which helps the fetal well-being monitoring and management of pregnancies complicated by hypertensive disorders or FGR [19, 20]. Most importantly, we first reported the hemodynamic changes associated with UAT with an emphasis on quantitative indices. Under normal circumstances, UA diastolic blood flow reflects the wall elasticity and distal resistance [21]. It has previously been reported that absent or reduced end-diastolic velocity (AREDV), among children born before 32 weeks of gestation, was associated with more frequent moderate or severe neuromotor or sensory disabilities [22, 23].

Compared with existing literature, Elena et al. [24]. analyzed 62 pregnant women with isolated single umbilical artery (SUA). Their data showed umbilical Doppler PI values in cases with SUA were about 20% lower than those expected for normal fetuses. In our data, we were surprised to find that UAT fetuses had no significant abnormal umbilical artery systolic peak compared with normal fetuses, but diastolic blood flow velocity was higher than controls, as a result the PI and RI values of UA were relatively lower than those in controls. Although 18.4% of fetuses were finally diagnosed with IUGR, Doppler ultrasound did not show AREDV, but increased diastolic blood flow. Similar to Elena’s finding, this surprising data suggested that in UAT fetuses, the mechanism of hemodynamic derangement could be due to the remaining artery to compensate hemodynamically, which caused increased diastolic blood flow and decreased PI. This mechanism also explains the formation of IUGR in our case may due to a lack of compensatory flow in the remaining artery.

Meanwhile, our data also presented that 44.7% (34/76) case combined with the redistribution of cerebral blood flow, characterized by increased middle cerebral artery diastolic flow and decreased MCA-PI. This change in diastolic blood flow may be due to dilation of blood vessels in the brain caused by the fetal self-protection effect. It is different from previous studies on the “brain protective effect” in fetuses [25], only 7 fetuses in our study with CPR abnormalities. We analyzed the unexpected result and found that it could be because both MCA-PI and UA-PI were decreased, the CPR within the normal range. As a consequence, it suggests that we should make a comprehensive assessment by combine MCA-PI and CPR, so as not to overlook “hidden brain protective effect” in MCA blood flow.

In addition, we categorized the distribution of blood flow abnormalities. Our relatively large sample data suggested that the redistribution of blood flow in the UA, MCA and DV is not a gradual change process. The interesting finding is that, fourteen UAT fetuses showed normal UA hemodynamic results, but other important hemodynamic changes were observed: 9 cases showed MCA-PI abnormalities only, and 5 cases showed MCA and DV abnormalities. Based on this, we conclude that the Doppler evaluation of MCA and venous system should not be abandoned in UAT fetuses when the UA blood flow value within the normal range, the blood flow re-distribution may have been occurred inadvertently.

*Perinatal management of UAT.* Treatment of UAT always in “dilemma” situation, especially in fetuses with small GA. More than one-third of the 76 fetuses were complicated by adverse outcomes (40.8%), proved that how to extend the pregnancy, accurately monitor the intrauterine situation and avoid adverse outcomes is particularly urgent and practical. For near-term fetuses in this study, 14 cases received urgent cesarean section, the mean GA at diagnosis was more than 32 weeks. Timely caesarean delivery may avoid intrauterine stillbirth or distress to some extent.

Slightly different from previous studies, our analysis focused on the time of pregnancy termination, which is crucial important to improve newborn quality. After the comprehensive analysis, the result showed that DV-PIV was the risk factor for poor prognosis. The parameters of DV was the direct reflection of the pressure and function of the right atrium [26]. In the updated guidelines, it was also mentioned that for high-risk fetuses, the blood flow of DV was also the focus, in addition to UA and MCA blood flow monitoring [12]. We observed 19 fetuses with decreased a-wave of intravenous catheter, 7 of which had stillbirth. All of these cases of intrauterine death occurred with DV-PIV below the 5th centile, and it is plausible that the DV-PIV play an indicator of fetal terminal stage combined with right atrial dysfunction.

Our cases also confirmed this conclusion that, although some UAT fetuses were detected in first or earlier second trimester or combined with UC abnormalities, after standardized Doppler evaluation and prenatal consultation, the rate of intrauterine death were significantly lower than previously reported [27]. Meanwhile, the median day between GA at diagnosis and end of pregnancy was more than previous researches. Surprisingly, UC abnormalities were not a risk factor for UAT in our study. The proportion of umbilical cord abnormalities was measured after delivery, not before. UC abnormalities were often missed during the prenatal period because of fetal position and the experience of the examiner.

The main limitation of our study was that a larger sample is needed to build predicting models for adverse pregnancy outcomes though the Doppler parameters and clinical features. Although there was known underlying placenta and umbilical cord etiology for UAT, the development of hemodynamic in these fetuses is likely multifactorial and deserves further investigation. Focus on this, the next stage of research is attempt to characterize the natural history of UAT throughout gestation and postnatally.

## Conclusions

Our data showed serious adverse pregnancy consequences are combined with UAT fetuses. Hemodynamic changes in UAT fetuses showed the remaining artery for compensation and brain perfusion derangement. With a comprehensive and standardized Doppler evaluation, progression of fetal deterioration may be detailed presented.

### Electronic supplementary material

Below is the link to the electronic supplementary material.

Supplementary Material 1

## Data Availability

The data that support the findings of our study are included in our article and additional supporting files. The raw data in this study are not publicly available in order to protect participant confidentiality, but are available from the corresponding author on reasonable request. If you want to request access to the data, please contact Prof. Suzhen Ran (ransuzhen0000@163.com) at Department of Ultrasound, Chongqing Health Center for Women and Children, Chongqing, China.
