# Supplementary material for: Hemodynamic changes and perinatal outcome associated with umbilical artery thrombosis: a retrospective study
Source: Orphanet J Rare Dis. 2024 Mar 5;19:100. doi: 10.1186/s13023-024-03107-y (PMC10913202; doi:10.1186/s13023-024-03107-y)
Supplement: Supplementary file 1 — Supplementary Material 1 [file 13023_2024_3107_MOESM1_ESM.docx]

Dear reviewer,

Thank you very much for your review and professional suggestions. These advise help to improve

academic opinion of our study, and it has important implications for our subsequent series of studies.

Based on your suggestion, we have made additional experiments and corrections. At the same time, we tried my best to correct the language errors. After your review again, if there are still problems in the language, we will ask for professional services. Now, we would like to show the details as follows:

Reviewer #1: General

1. Why have controls been selected, instead of e.g. reference values for a given fetal age, as you do mention centile values in the discussion part of the paper? Even more relevant, how have these controls been selected (unclear to this reviewer)

Response: Corresponding adjustments to the methods and results of the article were made. The explanation for the adjustments as follows, hoping to make you better understanding, if there's anything else I haven't clarified, please let me know.

(1) At present, **the reference values for a given fetal age are mainly about PI and RI.** **Our most important purpose is to explore the root cause of changes in PI and RI, such as systolic peak, diastolic flow rate and other specific adjustments.** We used a **control group of fetuses** in the same period for comparison, which may be more comparable to the data. Based on these results, we can explore the adaptation principle of UAT fetal hemodynamic parameters. (2) In addition, in methods section, we added **the inclusion criteria of the control group according to your suggestion**. (3) We mention **centile values in the discussion part**. Methods section, **the object is** “To investigate the specific distribution of hemodynamics in UAT fetuses, all parameters were compared with reference indexes for gestation, and the distribution of blood flow abnormalities were categorized.” Discussion section, we concluded “the redistribution of blood flow in the UA, MCA and DV is not a gradual change process, the Doppler evaluation of MCA and venous system should not be abandoned in UAT fetuses when the UA blood flow value within the normal range, the blood flow re-distribution may have been occurred inadvertently”.

2. Have you considered to explore associations between the extent of hemodynamic changes and the subsequent perinatal outcome? Likely relevant, although the design does not allow causality assessment.

Response: Thank you very much for your suggestion on the correlation between hemodynamic changes and outcome of UAT fetus. We are in complete agreement with you. This correlation study is the most critical to the outcome of UAT fetus.

**In this complementary experiment, we analyzed data on the risk factors and predictors of adverse pregnancy outcomes in UAT fetuses**. After the comprehensive analysis, the result showed that DV-PIV was the risk factor for poor prognosis. DV-PIV had moderate predictive accuracy for adverse pregnancy outcomes (AUC [95% CI] 0.792 [0.668–0.917]; *p* < 0.001). The result also showed that UAT fetuses with a DV-PIV value more than 0.76 tended to have adverse pregnancy outcomes (Youden’s index was 0.673), with sensitivity of 71.4% and specificity of 95.8%.

The main limitation of our study was that a larger sample is needed to build predicting models for adverse pregnancy outcomes though the Doppler parameters and clinical features, this is our next step with UAT fetuses.

3.Was IUGR present before the thrombosis, or occurs only afterwards?

Response: This issue was overlooked in our previous studies, but it is very important for the management of UAT fetal with IUGR, so we supplemented this part in the result section, thank you for your crucial reminder. **The characteristics of UAT with IUGR presented in Table 3 separately.** A total of 14 UAT fetuses were associated with IUGR, 12 of which were detected at the same stage with diagnosis, one was 45 days after diagnosis of UAT and one was 30 days before diagnosis of UAT.

4. Were all cases 'asymptomatic' at presentation (like fetal wellbeing, activity, biophysical profile)?

Response: **Fetal heart tracing** was performed on 65 pregnant women, the minority of pregnant women in UAT group (10/65, 15.4%) had non contractile stimulation test (NST) abnormalities. **In addition, a total of 14 fetuses developed to FGR.** Prenatal management of UAT fetuses is tricky for pregnant women and obstetricians, and the rate of fetal movement abnormalities is not as high as expected, so we included statistics on NST abnormalities in our initial design. However, only a small number of fetuses received biophysical scores because of comprehensive ultrasonic Doppler and fetal heart monitoring, so we didn't count **the results of the biophysical scores,** sincerely hoping to get your understanding.

5. Is there any exploration on the etiology of the thrombosis (maternal, fetal)? You only describe aspect related to length and torsion of the umbilical cord (could be relevant for the mother and subsequent pregnancies).

Response: Discussion section, **“The pathogenesis of UAT”** **was added**. In our study, UC abnormalities were present in a large proportion (36.8%), and the incidence of maternal complications such as GDH, GH, coagulation function abnormalities and other factors may also be the pathogenic factors for UAT. However, the data of the current study does not allow us to design causality assessment and risk prediction model, which is our next research design. Thank you very much for your approval.

6. I assume that prenatal 'lung' maturation was part of the management? if so, suggest to add

Response: I strongly agree with your suggestion. By consulting the medical electronic medical record system, obstetricians did perform lung maturation treatment before delivery in some cases. **Method section, we have added the part of promoting lung maturation.** “Low molecular weight heparin (LMWHs) and oxygen inhalation were used as appropriate, and **dexamethasone was given to accelerate fetal lung maturation**.” However, because the focus of this study was to discuss the mechanism of hemodynamic changes in UAT fetuses and the correlation with outcomes, we did not mention specific doses to promote lung maturation in the results part, sincerely hoping to get your understanding.

7. Can you add some information on the LMWH treatment (dose, target, monitoring?) and rationale, as this drug does not cross the placenta.

Response: Randomized trials have shown that LMWH may prevent recurrent placenta-mediated complications, such as preeclampsia (PE), fetal growth restriction (FGR), intrauterine fetal death (IUFD), and abruptio placentae, but available data are still conflicting. **We added the indication in Result section. During the management, LMWHs was used in the treatment of 7 pregnant women, the indications were 1 case of thrombophilia, 1 case of non-obstetric antiphospholipid syndrome (NC-OAPS), 2 cases of FGR, 1 case of FGR with NC-OAPS, 1 case of FGR with thrombophilia, and 1 cases of empirical use of LMWH to improve placental circulation.** Same as dexamethasone above, because the focus of this study was to discuss the mechanism of hemodynamic changes in UAT fetuses and the correlation with outcomes, we did not mention specific doses and monitoring in the results part. However, we know that the sample size of LMWH applied to UAT fetus is very limited. **If you still want to know the specific dose and monitoring, please let us know, and we can compile a supplementary table.**

Specific

1. The methods of the abstract is not explicit on the inclusion of only singletons, please check.

Response: The methods of the abstract was checked. **“We included singleton fetuses diagnosed with UAT and with at least one available Doppler evaluation before the end of pregnancy in our center from 2016 to 2023.”**

2. Results section abstract, the text suggests somewhat that the fetuses have 'chosen' their management. Highly recommend to rephrase this.

Response: The Results section abstract and results section were rephrased. **“After comprehensive prenatal counseling, 14 cases received urgent cesarean section, the remaining 55 cases received expectant management”.**

3. Overall, the language needs some additional verification and checking throughout the paper.

Response: We know that language is not what we are good at. We tried our best to revise the language, but during the major revision we mainly focused on revising the content of the article. **After the content has been approved, we will submit the issue of the language of the article to a professional organization.**

Reviewer #2: In this manuscript, the authors made a description of the hemodynamic changes and perinatal outcome in UAT fetuses with a relatively large sample.

Reviewer Comments:

1.UAT is a fairly rare disease, previous studies have shown an incidence of between 0.001% and 0.045%. In this study, eighty-nine singleton fetuses with UAT were identified in a single center from 2016 to 2022, the incidence rate (0.06%) was relatively high. Do the authors have an explanation for this?

Response: Thank you for your rigorous attitude towards the data in the article. We modified the data after checking. “**The incidence of UAT is 0.025% to 0.045%”.** In case series by Wei et al, the incidence of UAT was 0.8%, probably due to both our center were tertiary referral care center. And it was added in the discussion section.

2.In abstract section, the study period was "from 2016 to 2022". However, it was "from January, 2016 to June, 2023" in Method section. Do the authors have an explanation for this?

Response: We apologize for our handwriting errors and careless checking. In this overhaul, we checked all the data again. In abstract section, **"We included singleton fetuses diagnosed with UAT and with at least one available Doppler evaluation before the end of pregnancy in our center from 2016 to 2023".**

3.Methods section. The diagnostic clue for UAT were "two umbilical arteries were detected in the first or earlier second trimester by color Doppler and an absent paravesical color Doppler flow was seen in second- and third-trimester", which meaned that twice ultrasound scanning were necessary for the prenatal diagnosis of UAT. However, inclusion criteria for UAT was "with at least one fetal biometry and Doppler measurements until delivery available for review". Please recheck it.

Response: For the confusion caused to you by the inclusion criteria of UAT fetuses, we rechecked it. Methods section, **the diagnostic clue for UAT**: color flow mapping should be used to identify the image of the bladder with the two umbilical arteries in the first or earlier second trimester. And color Doppler flow present an absent paravesical during the next second-trimester ultrasound examination. **Further inclusion criteria for UAT**: **after the identification of UAT**, at least one fetal biometry and Doppler measurements available for review.

That means the diagnosis was confirmed by color flow mapping, and the hemodynamic evaluation was made by color Doppler flow, these two methods were used slightly differently. If there's anything else I haven't clarified, please let me know.

4.Methods section. "Control fetuses were GA-matched based on the time of the last fetal Doppler measurements", Doppler parameters can not be used for determining fetal GA. Biometric data measured by ultrasound are usually used for speculating fetal GA.

Response: So sorry for the confusion caused to you. Methods section, We rechecked it that “**The same sample of GA-matched normal fetuses with Doppler measurements during the same period were randomly selected as the control group”. Pregnancies were dated according to the last menstrual period (LMP) and confirmed by crown-rump length (CRL) measurements at 11-14week nuchal translucency scan.**

5.Results section. Changes of Doppler parameters should play the warning role to the adverse outcomes of fetuses with UAT. It is better to provide the cutoff values of Doppler parameters for predicting the adverse outcome, that would be of great significance in clinic practice. Please add this to the Results and Discussion sections.

Response: Thank you very much for your suggestion on the correlation between hemodynamic changes and outcome of UAT fetus. This correlation study is the most critical to the outcome of UAT fetus.

Results section. **In this complementary experiment, we analyzed data on the risk factors and predictors of adverse pregnancy outcomes in UAT fetuses**. After the comprehensive analysis, the result showed that DV-PIV was the risk factor for poor prognosis. DV-PIV had moderate predictive accuracy for adverse pregnancy outcomes (AUC [95% CI] 0.792 [0.668–0.917]; *p* < 0.001). The result also showed that UAT fetuses with a DV-PIV value more than 0.76 tended to have adverse pregnancy outcomes (Youden’s index was 0.673), with sensitivity of 71.4% and specificity of 95.8%.

Discussion sections. **Perinatal management of UAT. The parameters of DV are the direct reflection of the pressure and function of the right atrium**. In the updated guidelines, it was also mentioned that for high-risk fetuses, the blood flow of DV was also the focus, in addition to UA and MCA blood flow monitoring. We observed 19 fetuses with decreased a-wave of intravenous catheter, 7 of which had stillbirth. All of these cases of intrauterine death occurred with DV-PIV below the 5th centile, and it is plausible that the DV-PIV play an indicator of fetal terminal stage combined with right atrial dysfunction.

6.Discussion section. "The incidence of UAT is 0.0025%-0.045%", please recheck the data.

Response: Thank you for your rigorous attitude towards the data in the article. We have modified the data after checking. “**The incidence of UAT is 0.025% to 0.045%”.**

7.Discussion section. It is better to closely focus on the results of the MS in the discussion section.

Response: We are so sorry that **we did not find the true meaning of "MS" in the original text.** Please tell us the meaning of "MS". We sincerely look forward to your reply.

8.At the end of the paper, it seems that the "conclusion" section was absent.

Response: Thank you very much for your suggestion. **We added the conclusion section at the end of the paper.**

9.There are lots of mistakes in English expressions, please revise them sentence by sentence.

Response: We know that language is not what we are good at. We tried our best to revise the language, but during the major revision we mainly focused on revising the content of the article. **After the content has been approved, we will submit the issue of the language of the article to a professional organization.**
